# Longitudinal validity of using digital hand photographs for assessing hand osteoarthritis progression over 7 years in community-dwelling older adults with hand pain

**DOI:** 10.1186/s12891-019-2829-0

**Published:** 2019-10-27

**Authors:** Michelle Marshall, Helgi Jonsson, Gudrun P. Helgadottir, Elaine Nicholls, Helen Myers, Victoria Jansen, Danielle van der Windt

**Affiliations:** 1 Arthritis Research UK Primary Care Centre, School of Primary, Community and Social Care, Keele University, Keele, Staffordshire ST5 5BG UK; 2 Department of Rheumatology, Landspitalinn University Hospital, University of Iceland, IS-108 Fossvogur Reykjavik, Iceland; 3 University of Iceland, Reykjavik, Iceland; 4 Pulvertaft Hand Centre, Royal Derby Hospital, Derbyshire, DE22 3NE UK

**Keywords:** Hand joints, Osteoarthritis, Photography, Longitudinal studies, Radiographic, Outcomes, Physical examination, Clinical features

## Abstract

**Background:**

To determine the longitudinal construct validity of assessing hand OA progression on digital photographs over 7 years compared with progression determined from radiographs, clinical features and change in symptoms.

**Methods:**

Participants were community-dwelling older adults (≥50 years) in North Staffordshire, UK. Standardized digital hand photographs were taken at baseline and 7 years, and hand joints graded for OA severity using an established photographic atlas. Radiographic hand OA was assessed using the Kellgren and Lawrence grading system. Hand examination determined the presence of nodes, bony enlargement and deformity. Symptoms were reported in self-complete questionnaires. Radiographic and clinical progression and change in symptoms were compared to photographic progression. Differences were examined using analysis of covariance and Chi-Square tests.

**Results:**

Of 253 individuals (61% women, mean age 63 years) the proportion with photographic progression at the joint and joint group-level was higher in individuals with radiographic or clinical progression compared to those without, although differences were not statistically significant. At the person-level, those with moderate photographic progression over 7 years had significantly higher summed radiographic and clinical scores (adjusted for baseline scores) compared to those with no or mild photographic progression. Similar findings were observed for change in symptoms, although differences were small and not statistically significant.

**Conclusion:**

Assessing hand OA on photographs shows modest longitudinal construct validity over 7 years compared with change in radiographic and clinical hand OA at the person-level. Using photographs to assess overall long-term change in a person with hand OA may be a reasonable alternative when hand examinations and radiographs are not feasible.

## Background

Despite the high prevalence of hand osteoarthritis (OA) and the significant impact it has on pain experienced and interference with hand function little is still known about the course of the condition over time [1, 2].

Photographs of the hands have been used in the assessment of OA in a number of studies [3–8] in an attempt to provide an alternative method of classifying OA. Some of these early methods were insensitive to radiographically defined change [6, 7]. In 2012, the Age, Gene/Environment Susceptibility – Reykjavik (AGES-Reykjavik) photographic atlas was developed as a formal method for scoring hand photographs for OA [9]. It has been shown to be reliable and associated cross-sectionally with radiographic and clinical OA [10, 11] and shows age-related prevalence trends that are comparable to those seen for clinical and radiographic OA [12]. However, it is not known whether this method is valid in comparison to the progression of radiographic OA and the increased presence of clinical features determined from a physical examination.

Photographic assessment of hand OA, which does not require repeated radiation exposure, reduces research clinic time and costs, and decreases inter-observer variation through having a single central reader, would be extremely useful if validated. It will allow longitudinal epidemiological studies to be undertaken in large samples, over wide and remote geographic areas, and also could be of benefit for clinical monitoring of the condition especially in the provision of remote health care using telemedicine.

The aim of this study was to determine the longitudinal construct validity of assessing hand OA progression on digital photographs over a period of 7 years compared with hand OA progression determined from radiographs and from a hand examination of the clinical features over the same period. Additionally, hand OA progression on digital photographs will be examined to determine whether it is related to change in symptoms over 7 years.

## Methods

### Study population

The Clinical Assessment Study of the Hand (CASHA) is a prospective observational cohort study. All individuals aged 50 years and over from two general practice registers in North Staffordshire, UK were invited to participate in a two-stage postal survey that collected data on demographics and characteristics of hand pain and hand problems in 2004–5. Respondents who indicated that they had experienced hand pain or hand problems in the last year were invited to attend a research clinic. Individuals who attended the baseline research clinics were contacted again at 7 years (2011–12) and invited to complete a postal questionnaire and attend a follow-up research clinic. Research clinics at both time points included: digital photographs of the hands, radiographs of the hands and a physical hand examination [13]. Seven years was deemed a suitable time period over which some structural progression and soft tissue changes would be seen in a community-dwelling population. Questionnaires at both time points included the Australian/Canadian Hand Osteoarthritis Index (AUSCAN) pain, function and stiffness subscales [14] and at 7 years a question about the perceived change in their hand problem since baseline [15]. The study was approved at baseline by North Staffordshire Local Research Ethics Committee (reference: 1430) and at 7-years by West Midlands National Research Ethics Service (reference: 11/WM/0196), and all participants provided written informed consent.

### Digital hand photography

**Fig. 1:**
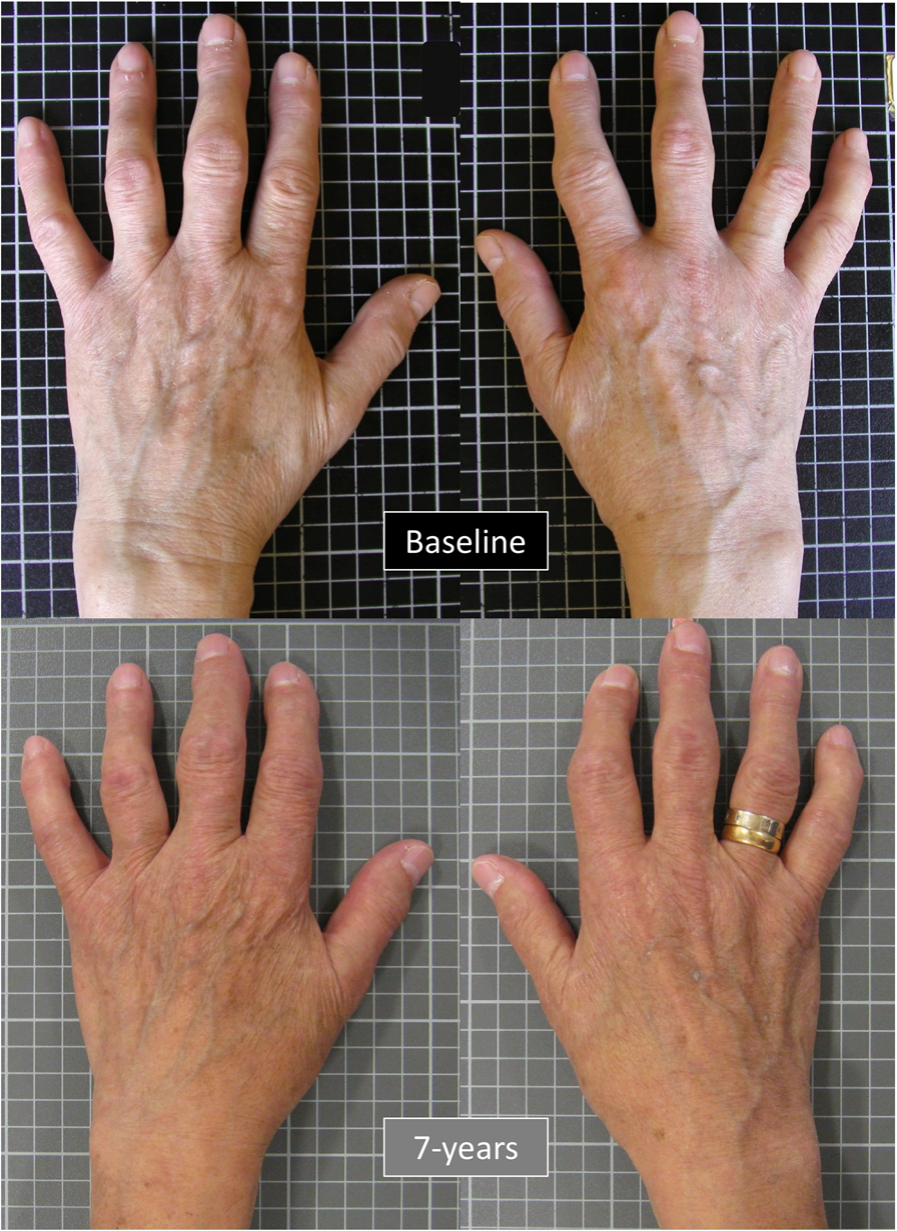
An example of the photographic hand images obtained at baseline and 7 years for a participant

Posterior digital photographs were taken of each hand separately using at baseline an Olympus Camedia C-4040 (Resolution 3.8 megapixels (MP)) and at follow-up either a Canon IXUS75 (resolution 7.1MP) or a Canon Powershot A480 (resolution 10.0MP). Hand photographs at both time points were collected in the same standardised position with the camera placed in a fixed position 38 cm (15 in.) above a gridded stand and were comparable to those used in the AGES-Reykjavik photographic scoring system [9]. The participants were seated with their shoulder adducted and the elbow flexed to 90^O^. The hand was pronated and placed in a fixed position on the gridded stand with forearm, wrist and fingers in a straight line and the hand resting in a natural position, i.e. with the fingers and thumb not held closely together or spanned. Figure 1 provides an example of the images taken at baseline and 7 years for a participant.

An experienced reader (HJ) who was blinded to the clinical and radiographic data graded photographs from baseline and 7 years using the AGES-Reykjavik photographic scoring system. As per the scoring system, the presence and severity of hand OA were graded on unpaired photographs but with a known time sequence (chronological) [9]. Five joints in each hand (second and third distal interphalangeal (DIP), second and third proximal interphalangeal (PIP) and first carpometacarpal (CMC) joints were examined visually for the presence of hard tissue (bony) enlargement, deformity and nodes. While OA is not restricted to these joints, for feasibility they were selected for examination in this study as they are the joints assessed in the clinical ACR hand OA criteria. Each joint was given a score on a 0–3 scale, with the assistance of a reference photographic collection, where 0 = normal: no evidence of OA, 1 = mild: some evidence of OA but not fulfilling the criteria for definite disease, 2 = definite: moderate OA and 3 = severe OA. Joint groups across both hands (DIP, PIP and CMC joints) were also graded using the same 0–3 scale with the highest scoring joint determining the grade for a joint group. Hand OA on digital photographs was defined as being present in a joint or joint group if the grade was ≥2.

For the purpose of assessing reliability, a second experienced reader (GPH), also blinded to the clinical and radiographic data and the scores of the first reader, scored all the 7-year hand photographs (*n* = 253) to determine inter-rater reliability. Intra-rater reliability was determined by (HJ) reading a sample of the hand photographs (*n* = 30) for a second time after at least four weeks.

### Radiographic scoring

Dorsipalmar (DP) radiographs of the hands and wrists were taken at baseline and 7-year follow-up according to a standardised protocol [13]. A single reader (MM), blinded to all questionnaire, clinical assessment and photographic data, graded all the baseline and 7-year hand radiographs unpaired with known chronological order. The presence and severity of OA using the Kellgren and Lawrence (KL) grading scale (0–4) was used to assess the second and third DIP, second and third PIP and first CMC joints in each hand [16].

Intra-rater reliability for the presence of hand OA in a joint was found to be excellent (mean Kappa (K) = 0.85 and percentage exact agreement (PEA) = 95%). Inter-rater reliability had previously been established with a second reader, an academic rheumatologist (RD) and found to be good (mean K = 0.79 and PEA = 95%) [17].

### Physical examination

Physical examination of the hands was undertaken at baseline and follow-up research clinics by trained physiotherapists and occupational therapists. The presence of nodes, hard tissue (bony) enlargement and deformity was determined in the second and third DIP, second and third PIP and first CMC joints in each hand and also any swelling present in the metacarpophalangeal (MCP) joints were recorded. Whilst we did not undertake formal reliability testing of the physical assessment in the study at baseline and 7 years, quality assurance and control were integral parts of the study as detailed in the study protocol [13]. Clinical assessors, qualified occupational and physiotherapists, underwent training for the physical assessment techniques. A detailed Assessors Manual with protocols for physical assessment was provided to assessors for reference during the study and quality control sessions were undertaken at regular intervals throughout the study. Therapists were not aware of the photographic or radiographic scores as these were determined after the research clinics had taken place.

### Exclusions

Participants were excluded from this analysis if they did not have digital hand photographs at both baseline and 7-year follow-up and if general practice or local rheumatology medical records or a musculoskeletal radiologist identified them as having inflammatory arthritis (rheumatoid or psoriatic arthritis) at baseline. They were also excluded if there was an indication of possible inflammatory arthritis or other serious pathology (scleroderma, neuropathic changes or severe contracture) on the digital hand photographs at baseline or 7-year follow-up, as determined by a consultant rheumatologist (HJ). Additionally, if a participant reached the maximum score at baseline for the photographic, radiographic scores or the maximum number of clinical features, then they were excluded from selected joint and person-level analyses as they were unable to progress.

### Statistical analysis

Reliability of using the photographic scoring system has previously been established [11], thus reliability was undertaken at 7-years to ensure that this hadn’t markedly changed. The inter-rater reliability of the scoring of photographic hand OA at 7 years was assessed across all participants using intra-class correlation coefficients (ICC), using 2-way random effects models with absolute agreement, for the 10 hand joints and three joint groups. Intra-rater reliability was also assessed, to ensure consistency of scoring by the main observer (HJ), in a randomly selected sample (*n* = 30) using ICCs for the three joint groups.

No reference standard is available for assessing progression on digital hand photographs so examining criterion validity was not possible. Therefore, longitudinal construct validity was investigated against a number of constructs. At the joint and joint group level the proportion of individuals who had shown photographic hand OA progression deom baseline to 7 years (increase in score ≥ 1) was calculated for individuals i) with (increase in score of ≥1) and without (change in score of < 1) radiographic progression between baseline and 7 years and ii) with (increase in score of ≥1) and without (change in score of < 1) progression of clinical features between baseline and 7 years. For a joint group, the highest change in in any joint between baseline and 7 years that was present within a joint group was used for photographic OA, radiographic OA and the number of clinical features. In addition, change scores were also calculated to represent the magnitude of change at each joint and in each joint group (7-year follow-up score – baseline score) for each construct (photographic, radiographic, clinical features). As data were not normally distributed, Spearman Rank correlation coefficients were used to assess how similar the constructs of radiographic OA and number of clinical features present were to the hand photographic scoring. Two a priori hypotheses were set regarding these correlations at the joint and joint group level:

1) Change in photographic hand OA scores for each joint was expected to correlate more closely with the change in the number of clinical features than with the change in radiographic OA grade. 2) Correlations would be the same or slightly lower than was achieved at baseline for cross-sectional construct validity in each hand joint and joint group [11].

At the person-level photographic hand OA scores were summed for the 10 assessed hand joints and change scores calculated (7 year – baseline; max range − 30 to 30). Individuals were divided into tertiles based on the distribution of data and categorised into those that had not progressed (change score ≤ 0), those that had undergone mild progression (change score = 1 to 2) and those that had undergone at least moderate progression (change score ≥ 3). Additionally, the number of joints that had undergone photographic hand OA progression over 7 years was examined using tertiles based on the distribution of data and categorised into no progression (0 joints), mild progression (1–2 joints) and at least moderate progression (≥3 joints). Analysis of variance was used to describe the mean (and 95% confidence intervals (CI)) summed radiographic OA score at 7-years, summed number of clinical features at 7-years, and 7-year AUSCAN pain (0–20), function (0–36), stiffness (0–4) and total (0–60) scores adjusted for baseline score for each tertile and to test for significance of differences between the tertiles and determine if change over time was similar between the different constructs. Percentages and Chi-Square Tests were used to explore differences in individuals perceived global assessment of change in their hand problem over 7-years (improved, no change, deteriorated) between the tertiles.

## Results

### Study population

From the 432 eligible participants invited to take part at 7 years, a total of 376 participants completed the questionnaire (response rate = 87.0%) and 256 of whom attended the 7-year research clinics (response rate 59.3%) (Additional file 1: Figure S1). Following exclusion for the absence of 7-year digital hand photographs and possible inflammatory arthritis, 253 individuals with paired baseline and 7-year digital hand photographs were included in the analysis. At the joint-level, up to 12 joints for photographic score, 28 joints for radiographic scores and 38 joints for clinical features were excluded due to having maximum baseline scores depending on the joint examined. At the person-level, *n* = 2 were excluded due to having the maximum baseline summed photographic hand OA score of 9 and *n* = 4 for having all ten hand joints affected with photographic hand OA at baseline.

Individuals followed-up at 7 years with repeat digital hand photographs compared to those without repeat hand photographs (*n* = 305) were: slightly younger; less likely to have a manual occupation; thumb pain during activity in the last month; photographic hand OA at baseline; and moderate to severe radiographic OA (KL ≥ 3) in one or more hand joints at baseline; but were more likely to have attended further education (Table 1).

**Table 1:** Baseline descriptive characteristics of the study participants

Baseline Characteristic	All eligible baseline participants (*n* = 558)	Participants without repeat digital hand photos at 7 years (*n* = 305)	Participants with repeat digital hand photos at 7 years (*n* = 253)
% Female (no.)	61.6 (344)	62.6 (191)	60.5 (153)
Age range, years	51–91	51–91	51 to 80
Mean age, years (s.d.)	64.2 (8.2)	65.4 (8.8)	62.7 (7.0)
Mean BMI (s.d.)	28.2 (4.8)	28.7 (4.9)	27.7 (4.6)
% Attended further education (no.)	16.2 (89)	13.1 (39)	19.8 (50)
% Manual occupational class (no.)	52.3 (274)	57.2 (162)	46.7 (112)
% Right-handed (no.)	90.8 (504)	89.8 (274)	90.9 (230)
% Hand pain or problems in the last month (no.)	85.8 (479)	87.5 (267)	83.8 (212)
% Thumb pain during activity in the last month % (no.)	53.0 (296)	56.4 (172)	49.0 (124)
Duration of hand symptoms % (no.):			
< 1 year	10.3 (53)	10.3 (29)	10.2 (24)
1–5 years	42.3 (218)	41.8 (118)	42.4 (100)
> 5–10 years	22.5 (116)	21.3 (60)	23.7 (56)
> 10 years	24.9 (128)	25.5 (72)	23.7 (56)
Summed photographic hand OA score for the 3 joint groups % (no.):			
0	42.6 (229)	38.2 (112)	48.0 (117)
1	18.8 (101)	19.8 (58)	17.6 (43)
2	15.1 (81)	14.0 (41)	16.4 (40)
3	8.9 (48)	9.9 (29)	7.8 (19)
≥ 4	14.5 (78)	18.1 (53)	10.2 (25)
Clinical hand OA % (no.):			
ACR criteria	29.6 (165)	29.8 (91)	29.4 (74)
Relaxed ACR criteria*	51.0 (284)	51.8 (158)	50.0 (126)
Radiographic OA % (no.):			
KL ≥ 2 in ≥1 joint	76.5 (427)	78.4 (239)	74.3 (188)
KL ≥ 3 in ≥1 joint	35.8 (200)	40.3 (123)	30.4 (77)

* Relaxed ACR criteria is when there is pain on some, most or all days rather than most days or all days in the ACR criteria. s.d., standard deviation; *BMI*, Body mass Index; *ACR*, American College of Rheumatology; *KL*, Kellgren and Lawrence grading system

#### Hand OA progression

**Fig. 2:**
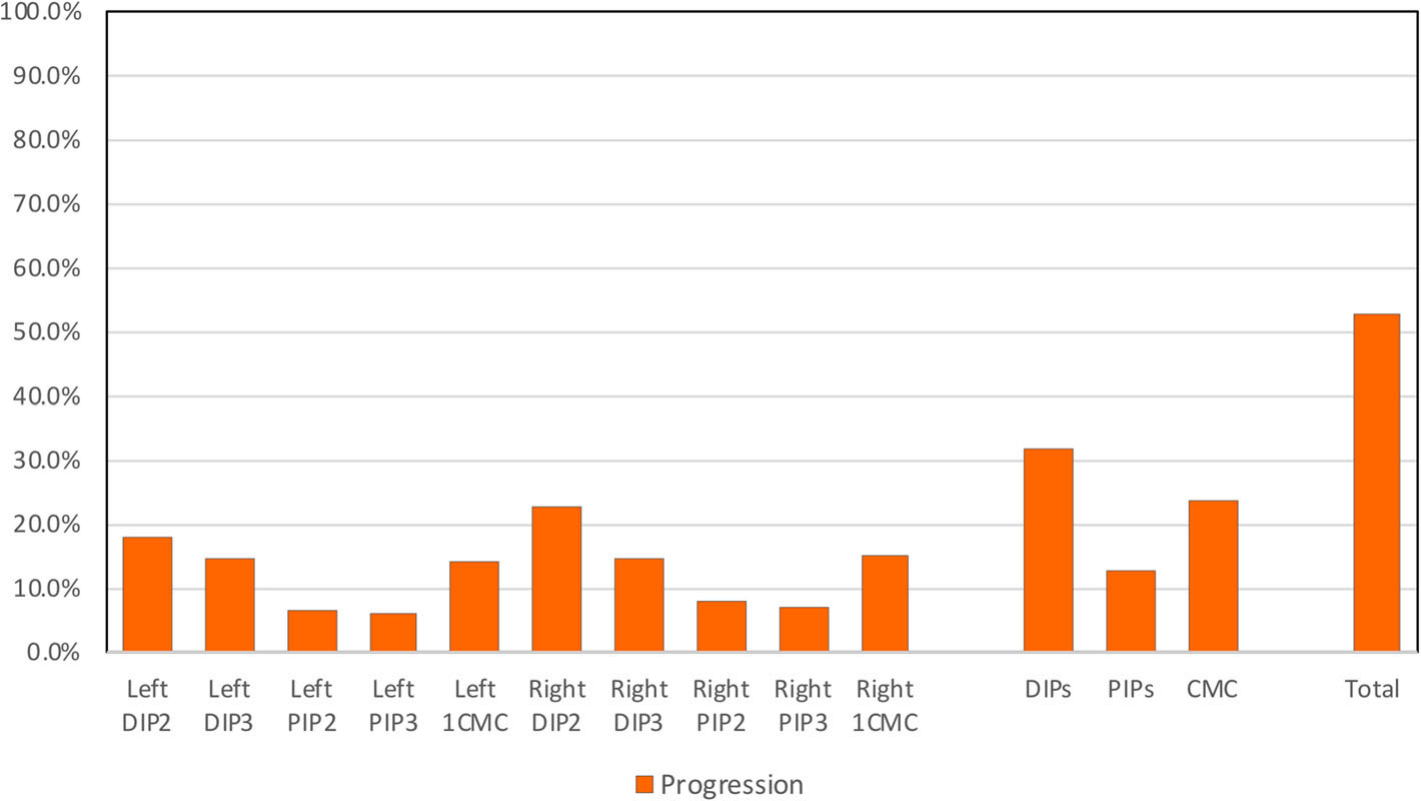
The amount of photographic hand OA progression over 7 years

Overall 52.6% (*n* = 133) of participants experienced photographic progression in one or more hand joints over 7 years. Progression on the hand OA photographs was seen in each joint, but occurred more often in the DIP joints (14.4–22.6%) than the CMC (14.2–15.2%) and PIP joints (6.1–7.8%) (Fig. 2).

### Reliability

The 7-year inter-rater reliability of the photographic hand OA scoring in all 253 participants was good to excellent (ICC = 0.65–0.95) for all hand joints and most joint groups, but moderate for the PIP joint group (ICC = 0.54) (Table 2). Intra-rater reliability undertaken on a subsample of 30 participants for the joints groups was excellent (ICC = 0.86–0.94).

**Table 2:** Reliability for scoring photographic hand OA by joint and joint group in all 253 participants

			ICC†	% exact agreement	% close agreement
Inter-rater	Right	DIP3	0.89	83.3	100
		DIP2	0.83	71.0	99.6
		PIP3	0.84	88.3	99.6
		PIP2	0.65	85.1	99.6
		1CMC	0.88	84.4	100
	Left	DIP3	0.85	81.9	100
		DIP2	0.80	70.8	98.6
		PIP3	0.82	90.2	100
		PIP2	0.80	91.4	99.6
		1CMC	0.82	81.3	99.6
	Joint group	DIPs	0.65	69.7	92.7
		PIPs	0.54	85.0	94.9
		1CMCs	0.95	93.7	100
Intra-rater	Joint group	DIPs	0.86	85.7	100
		PIPs	0.94	96.4	100
		1CMCs	0.90	92.6	100

† Intraclass correlation coefficients (ICCs) were calculated for absolute agreement using 2-way random-effects model for single measures. *DIP*, Distal Interphalangeal joint; *PIP*, Proximal Interphalangeal joint; *1CMC*, 1st Carpometacarpal joint

### Joint-level associations with photographic hand OA progression over 7 years

**Fig. 3:**
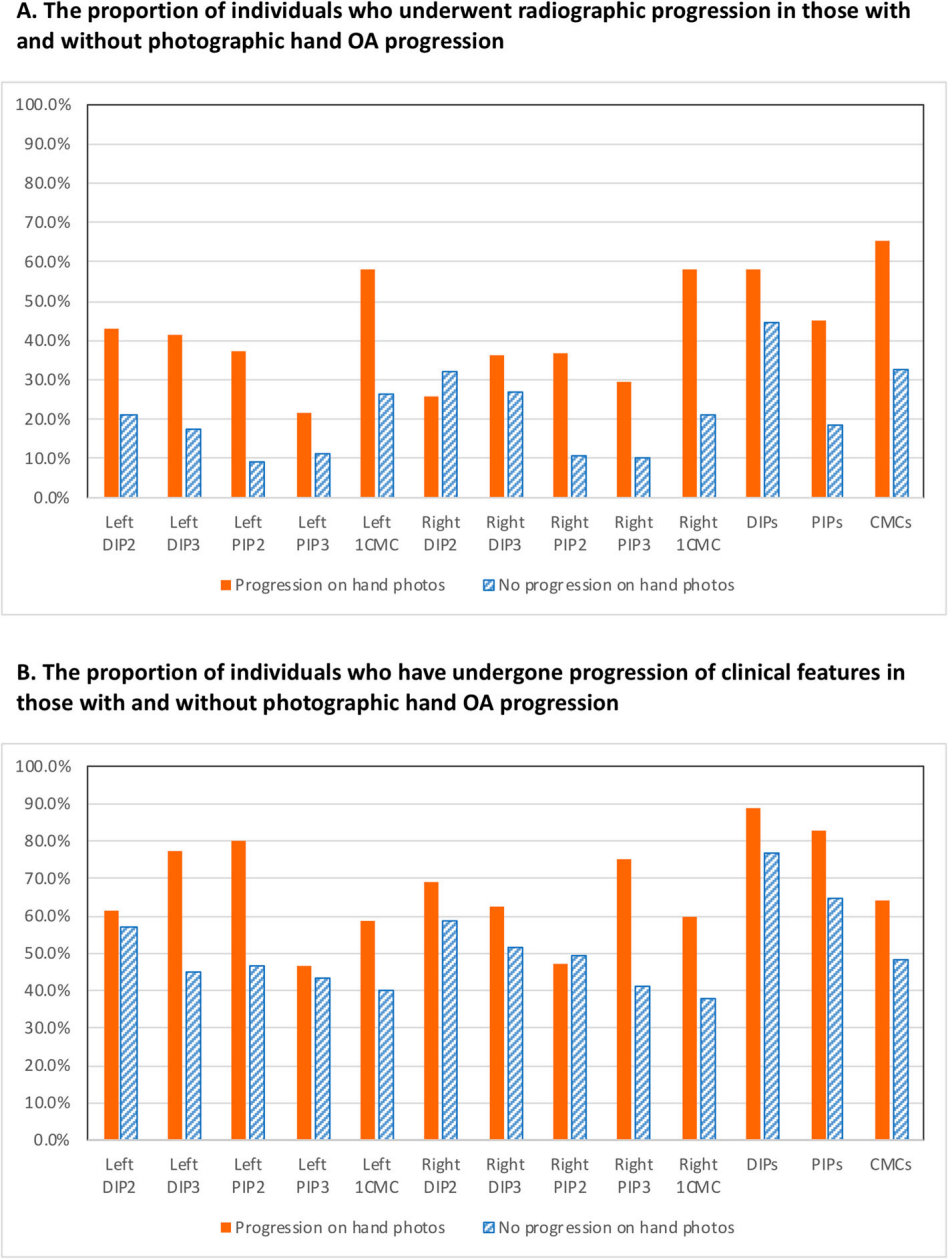
The proportion of individuals who underwent hand photographic progression in: **a**. those with and without radiographic hand OA progression. **b**. those with and without progression of clinical features

Overall the proportion of individuals who had experienced photographic hand OA progression over 7 years at the joint and joint group level were higher in those with radiographic hand OA progression compared to those without, however differences were small ranging between 6 and 22% (Fig. 3 A). The proportion of individuals who had experienced photographic hand OA progression over 7 years at the joint and joint group level were higher in those with an increase in the number of clinical features compared to those without, but again differences were smaller varying from 1 to 16% (Fig. 3 B). The only exceptions to this were the right index DIP (DIP2) for radiographic OA and the right index PIP (PIP2) for clinical features, where a higher proportion of participants with radiographic change or clinical features was found in those who showed no progression on hand photos.

Correlations between change in photographic hand OA and i) change in radiographic OA and ii) the change in the number of clinical features for joints and joint groups were mostly weak, although some were still statistically significant (Table 3). Change in photographic hand OA generally did not correlate more closely with the change in clinical features than with radiographic change as was hypothesised. Correlations in each hand joint and joint group were, for the most part, lower than was achieved for construct validity at baseline [11].

**Table 3:** Correlations between change in photographic hand score and change in (i) radiographic score and (ii) number of clinical features between baseline and 7 years by joint and joint group

		Change in photographic hand OA with change in radiographic OA		Change in photographic hand OA with change in the number of clinical features	
		n*	Rho (*p* value)	n*	Rho (p value)
Left	DIP2	231	0.138 (*p* = 0.036)	228	0.032 (*p* = 0.627)
	DIP3	234	0.202 (*p* = 0.002)	243	0.161 (*p* = 0.012)
	PIP2	244	0.198 (p = 0.002)	250	0.116 (*p* = 0.066)
	PIP3	236	−0.089 (*p* = 0.171)	243	0.076 (*p* = 0.239)
	1CMC	191	0.130 (*p* = 0.072)	189	0.094 (*p* = 0.197) †
Right	DIP2	216	−0.066 (*p* = 0.337)	214	0.097 (*p* = 0.155)
	DIP3	228	0.070 (*p* = 0.294)	231	0.092 (*p* = 0.164)
	PIP2	236	0.134 (*p* = 0.039)	242	0.023 (*p* = 0.717)
	PIP3	231	−0.019 (*p* = 0.773)	235	0.138 (*p* = 0.035)
	1CMC	182	0.102 (*p* = 0.170)	177	0.063 (*p* = 0.405) †
Joint group	DIPs	205	0.109 (*p* = 0.119)	201	0.109 (*p* = 0.124)
	PIPs	226	0.143 (*p* = 0.032)	228	0.241 (*p* < 0.001)
	1CMCs	162	0.107 (*p* = 0.176)	161	0.042 (*p* = 0.596) †

* Study population n = 253 but individuals were excluded from analyses if at baseline they had the maximum score in a joint for either construct. † Change in the number of clinical features were − 2 to 2 as only two clinical features for the thumb were assessed at baseline (deformity, joint enlargement). Correlations presented are Spearman Rank correlation coefficients. *DIP*, Distal Interphalangeal joint; *PIP*, Proximal Interphalangeal joint; *1CMC*, 1st Carpometacarpal joint

### Person-level associations with photographic hand OA progression over 7 years

Individuals undergoing at least moderate amounts of summed photographic hand OA progression (change score ≥ 3) over 7 years had significantly higher i) mean scores for radiographic hand OA and ii) the number of clinical features at 7 years after adjustment for baseline scores compared to those with no (change score ≤ 0) and mild (change score 1–2) photographic progression (Table 4). Those undergoing at least moderate amounts of photographic hand OA progression also had more pain, functional limitation, stiffness at 7 years than those with no or mild photographic progression after adjustment for baseline scores, although differences were not significant. Individuals with mild and moderate photographic progression had larger proportions that felt their hand problem had deteriorated over 7 years compared to those with no photographic progression.

**Table 4:** Person-level associations between photographic hand OA progression and radiographic, clinical and symptomatic outcomes at 7 years

	Change in summed photographic hand OA score			
Mean (95% CI)	No Progression (change ≤ 0) (*n* = 153)	Mild Progression (change 1–2)	Moderate progression (change ≥ 3)	ANOVA *p* value
		(*n* = 59)	(*n* = 41)	
7-year summed radiographic OA score* (0–40)	7.2 (6.4, 8.0)	8.2 (6.9, 9.4)	10.4 (8.9, 11.9)	F = 7.1 *p* = 0.001
7-year summed number of clinical features* (0–28)	10.6 (9.8, 11.5)	10.9 (9.6, 12.2)	14.4 (12.7, 16.0)	F = 8.3 *p* = < 0.001
7-year AUSCAN Pain * (0–20)	6.2 (5.5, 6.8)	6.6 (5.7, 7.6)	7.1 (5.9, 8.2)	F = 1.0 *p* = 0.366
7-year AUSCAN Function* (0–36)	9.9 (9.0, 10.8)	10.8 (9.3, 12.3)	10.5 (8.7, 12.3)	F = 0.5 *p* = 0.586
7-year AUSCAN Stiffness* (0–4)	1.0 (0.9, 1.2)	1.0 (0.8, 1.2)	1.2 (0.9, 1.5)	F = 0.9 *p* = 0.407
7-year Summed total AUSCAN score* (0–60)	17.0 (15.5, 18.5)	18.4 (16.0, 20.9)	18.6 (15.7, 21.5)	F = 0.7 *p* = 0.481
Global perceived change in hand problem % (*n*):				Chi Square *p* value
improved	23.0% (*n* = 35)	8.6% (*n* = 5)	9.8% (*n* = 4)	Χ^2^ = 13.0 *p* = 0.369
no change	25.7% (*n* = 39)	27.6% (*n* = 16)	24.4% (*n* = 10)	
deteriorated	51.3% (*n* = 78)	63.8% (*n* = 37)	65.9% (*n* = 27)	
	Change in number of hand joints with photographic hand OA progression			
Mean (95%CI)	No Progression (no joints) (*n* = 120)	Mild Progression (1–2 joint)	Moderate progression (≥3 joints)	ANOVA *p* value
		(*n* = 92)	(*n* = 41)	
7-year summed radiographic OA score* (0–40)	6.5 (5.6, 7.4)	8.6 (7.8, 9.7)	10.3 (8.8, 11.8)	F = 10.9 *p* < 0.001
7-year summed number of clinical features* (0–28)	10.5 (9.5, 11.4)	11.0 (9.9, 12.0)	14.4 (12.8, 16.1)	F = 8.4 p0.001
7-year AUSCAN Pain* (0–20)	5.9 (5.2, 6.6)	6.8 (6.0, 7.6)	7.2 (6.0, 8.4)	F = 2.5 *p* = 0.081
7-year AUSCAN Function* (0–36)	9.5 (8.4, 10.5)	10.6 (9.3, 11.8)	11.6 (9.8, 13.4)	F = 2.3 *p* = 0.102
7-year AUSCAN Stiffness* (0–4)	1.0 (0.8, 1.1)	1.1 (0.9, 1.3)	1.2 (0.9, 1.5)	F = 1.1 *p* = 0.330
7-year Summed total AUSCAN score* (0–60)	16.1 (14.4, 17.8)	18.5 (16.5, 20.4)	19.8 (16.9, 22.7)	F = 2.9 *p* = 0.056
Global perceived change in hand problem % (*n*):				Chi Square *p* value
improved	22.7% (*n* = 27)	15.4% (*n* = 14)	7.3% (*n* = 3)	Χ^2^ = 13.3 *p* = 0.345
no change	27.7% (*n* = 33)	24.2% (*n* = 22)	24.4% (*n* = 10)	
deteriorated	49.6% (*n* = 59)	60.4% (*n* = 55)	68.3% (*n* = 28)	

* Adjusted for baseline score. *AUSCAN*, Australian/Canadian Hand Osteoarthritis Index; *ANOVA*, Analysis of Variance

Individuals who had ≥3 hand joints with photographic progression had significantly greater increases in radiographic OA and clinical features over 7 years compared to those with 1–2 joints or no joints that had progressed (Table 4). Individuals with ≥3 hand joints with photographic progression also had more pain, functional difficulties, stiffness at 7 years compared to those with 1–2 joints or no joints that had progressed after adjustment for baseline symptoms. While these differences showed a dose-response across the tertiles, the differences were not statistically significant.

## Discussion

This study is the first to investigate the longitudinal construct validity of assessing hand OA progression on digital photographs over a period of 7 years compared with progression determined on hand radiographs, clinical features on hand examination, and change in symptoms. We found that change in photographic assessment of hand OA at the person-level over 7 years does show longitudinal construct validity to changes in radiographic OA and clinical features. Individuals with the greatest amounts of photographic progression over 7 years had more pain and functional limitation than those with no or smaller amounts of progression, but differences were small. The results for longitudinal validity at the joint-level were less promising with weak correlations between changes in hand photographs and changes in radiographic OA or clinical features.

Overall we found that 51% of individuals progressed on their hand photographs over 7 years. While rates of progression have not been reported previously for photographic progression or even clinical features, the rate we obtained is comparable to the progression reported in other studies of a similar length for radiographic change (53% over 6 years [18]), change on Magnetic Resonance Imaging (MRI) (58% over 5 years [19]) and symptoms (49% over 4-years [20]).

At the person-level, those with moderate progression on the hand photographs had significantly higher scores for summed radiographic OA and clinical features compared with those classed as having none or mild progression. Moderate photographic progression in terms of summed change score and number of hand joints undergoing progression did also show stronger relationship to changes in symptoms, however, associations were non-significant. Change in the number of hand joints undergoing photographic hand OA progression seemed to be more sensitive than change in the summed photographic hand OA score. Mixed associations between symptoms and structural change have been reported in hand OA [18, 21] and it is possible this is due to hand OA frequently co-existing with other hand conditions [1]. While we excluded individuals with inflammatory arthritis, other individuals may have had other hand conditions such as carpal tunnel syndrome, Dupuytren’s contracture and trigger finger that could have contributed to the symptoms reported at both baseline and follow-up and weakened the associations we found.

Stronger relationships of photographic OA with radiographic OA and clinical features were seen at the person-level compared to the joint-level. While we found that overall about half of the individuals underwent any photographic progression in the hands, the numbers undergoing progression in the individual hand joints was much smaller (between 6.1–22.5). At the joint and joint group level, correlations between change in hand photographs and change in radiographic OA or clinical features were weak, but this was expected as the constructs were not similar. Another explanation of weaker correlation between photographic and clinical change is that only a single 2D photograph of the back of the hands was examined which may mean that some changes to the hands such as fixed flexion deformity could be missed. While this is similar to the single PA view taken radiographically it is possible that photographs taken from other views such as a lateral or even an oblique may be helpful and important for assessing clinical changes.

While we were able to examine longitudinal construct validity, it was not possible to examine responsiveness as there is no accepted measure of clinically important change in hand OA, and no reference group that had not undergone any OA progression. The mean changes seen over 7 years in our general population sample were small, and it is possible that progression rates in a more severe clinical sample might have offered a better opportunity to examine longitudinal validity, although this could have limited generalisability of findings.

We acknowledge a number of possible limitations of this study. Individuals that took part in the follow up at 7 years were less likely to have photographic hand OA at baseline than those without follow-up hand photographs, but we don’t envisage that this selective attrition is likely to have affected the relationships between the constructs within our study sample. Hand photographs were scored unpaired but with a known time sequence which has been suggested might lack sensitivity that could be obtained by reading in pairs and lead to an overestimation of change [22]. However, the changes we saw were only small, and reliability was good to excellent and the reading of the hand photographs unpaired with known order meant that it was comparable to the approaches taken to assess the clinical features of hand OA and the hand radiographs as part of the cohort study. To date, scoring of photographic images for OA using the AGES-Reykjavik scoring system has been limited to researchers who developed the atlas, further testing the reliability and validity should be undertaken by other investigators. Additionally, the longitudinal validity of a photographic grading system was compared to radiographic imaging which is known to be not as sensitive as ultrasound and MRI [23, 24] and does not allow the visualisation of the full range of joint tissues affected by hand OA [25–28]. Therefore other imaging methods, although more expensive and less feasible in large population-based studies, may have been more sensitive in determining structural change against which photographic change could have been compared. Additionally, other methods such as machine learning, which have been successfully applied to MRI images, may prove to be more sensitive in assessing hand photographs for the subtle differences that determines the development but also the progression of clinical features of OA [29].

## Conclusions

The assessment of photographic hand OA at the person-level over 7 years shows modest longitudinal construct validity in relation to radiographic OA and clinical features, but longitudinal validity at the joint-level was less favourable. Using hand photographs may be a reasonable method for examining overall change in a person with hand OA over the long term when radiographs or a hand examination is not feasible such as in large, populating-based studies or those undertaken over wide geographic areas.

## Supplementary information

**Additional file 1: Figure S1.** Flow diagram of study participants

## Data Availability

The dataset analysed during the current study is available on reasonable request. The Arthritis Research UK Primary Care Centre has an established data sharing arrangements to support joint publications and other research collaborations. Applications for access to anonymised data from our research databases are reviewed by the Centre’s Data Custodian and Academic Proposal (DCAP) Committee and a decision regarding access to the data is made subject to the ethical approval that was obtained. Further information on our data sharing procedures can be found on the Centre’s website (http://www.keele.ac.uk/pchs/publications/datasharingresources/) or by emailing the Centre’s data manager (primarycare.datasharing@keele.ac.uk).

## Abbreviations

ACR: American College of Rheumatology
ANOVA: Analysis of Variance
AUSCAN: Australian Canadian Hand Osteoarthritis Index
BMI: Body Mass Index
CASHA: Clinical Assessment Study of the Hand
CI: Confidence Interval
CMC: Carpometacarpal
DCAP: Data Custodian and Academic Proposal
DIP: Distal Interphalangeal
DP: Dorsipalmer
ICC: Intra-class Correlation Coefficients
K: Kappa
KL: Kellgren and Lawrence
MCP: Metacarpophalangeal
MP: Megapixels
OA: Osteoarthritis
PEA: Percentage Exact Agreement
PIP: Proximal interphalangeal
s.d.: Standard Deviation
