# Supplementary figures and images for: Longitudinal validity of using digital hand photographs for assessing hand osteoarthritis progression over 7 years in community-dwelling older adults with hand pain
Source: BMC Musculoskelet Disord. 2019 Oct 27;20:484. doi: 10.1186/s12891-019-2829-0 (PMC6815403; doi:10.1186/s12891-019-2829-0)

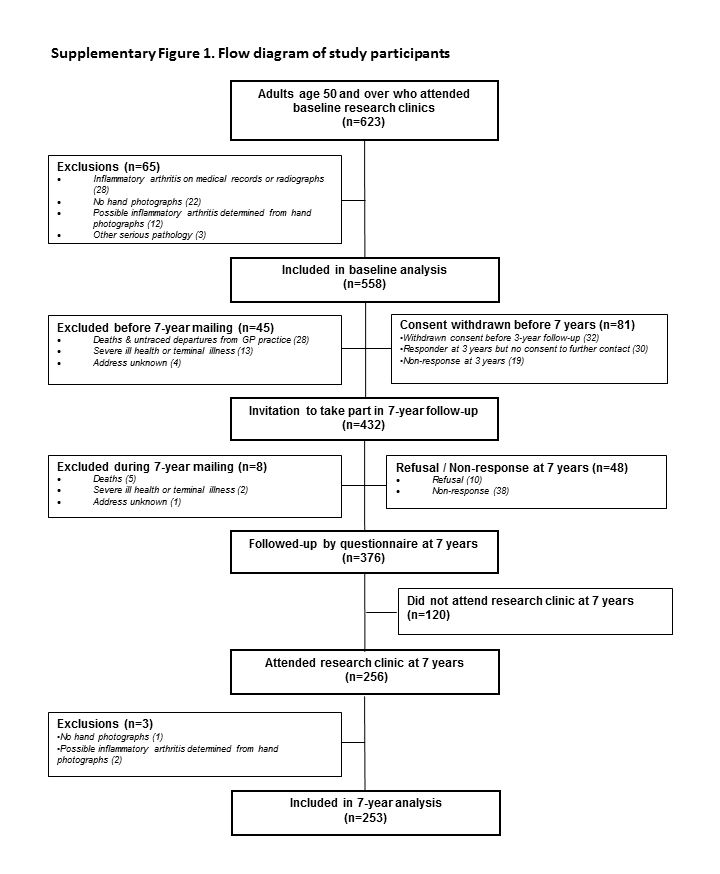

Supplement: Supplementary file 1 — Additional file 1: Figure S1. Flow diagram of study participants [file 12891_2019_2829_MOESM1_ESM.tif]
